# Astaxanthin Modulates Inflammation in Type 2 Diabetes via Regulation of microRNAs, Lysophosphatidylcholine, and α-Hydroxybutyrate

**DOI:** 10.1155/ije/5878361

**Published:** 2025-08-20

**Authors:** Ali Sharifi-Rigi, Fatemeh Zal, Mohammad-Hossein Aarabi, Mehdi Dehghani, Nikoo Roustaei Rad, Sana Taghiyar

**Affiliations:** ^1^Student Research Committee, Shiraz University of Medical Sciences, Shiraz, Iran; ^2^Department of Biochemistry, School of Medicine, Shiraz University of Medical Sciences, Shiraz, Iran; ^3^Department of Clinical Biochemistry, School of Pharmacy and Pharmaceutical Sciences, Isfahan University of Medical Sciences, Isfahan, Iran; ^4^Consultative Genomix, Houston, Texas, USA; ^5^Department of Clinical Biochemistry, International Campus, Shahid Sadoughi University of Medical Science, Yazd, Iran

**Keywords:** α-hydroxybutyrate, astaxanthin, inflammation, lysophosphatidylcholine, miRNAs, Type 2 diabetes

## Abstract

Astaxanthin is a carotenoid compound that has several beneficial qualities, including antioxidant, anti-inflammatory, antiapoptotic, and antidiabetic effects. This study examined the effects of astaxanthin supplementation on inflammation-related microRNAs, lysophosphatidylcholine, and α-hydroxybutyrate in individuals with Type 2 diabetes. Fifty people with Type 2 diabetes volunteered in a placebo-controlled, randomized, double-blind clinical trial. Subjects were randomly determined to consume either 10 mg of astaxanthin (*n* = 25) or a placebo (*n* = 25) for 12 weeks. Quantitative real-time PCR was employed to assess the expression of inflammation-related microRNAs in peripheral blood mononuclear cells both before and after the intervention, and we employed ELISA to ascertain the serum levels of lysophosphatidylcholine and α-hydroxybutyrate. After 12 weeks of supplementation, in comparison with placebo, astaxanthin supplementation resulted in a noteworthy decrease (*p* < 0.05) in hsa-miR-21, hsa-miR-34a, and hsa-miR-155 expression. In addition, astaxanthin supplementation substantially decreased (*p* < 0.05) the levels of lysophosphatidylcholine and α-hydroxybutyrate compared with the placebo. These changes suggest that astaxanthin may contribute to the modulation of inflammatory processes and the enhancement of metabolic homeostasis. Moreover, relative to the placebo, astaxanthin supplementation considerably diminished serum plasma glucose, HbA1c, lipid profile, and albumin-to-creatinine ratio levels. In conclusion, the current investigation indicates that astaxanthin supplementation at a dosage of 10 mg per day might be a useful strategy for ameliorating inflammation-related diabetic complications and insulin resistance in Type 2 diabetes patients. Considering the potential role of microRNAs in regulating inflammation and metabolic dysfunction in Type 2 diabetes, these findings suggest that astaxanthin supplementation may modulate inflammation-related microRNAs and metabolic markers, potentially contributing to the management of inflammatory processes and metabolic dysregulation in Type 2 diabetes.

**Trial Registration:** Iranian Registry of Clinical Trials (IRCT): IRCT20190305042939N1

## 1. Introduction

One of themost significant long-term challenges facing healthcare systems worldwide is the diabetes mellitus (DM) pandemic [1]. An oral glucose tolerance test (OGTT) value of 200 mg/dL or higher, fasting plasma glucose levels of 126 mg/dL or higher, or glycated hemoglobin (HbA1c) levels of 48 mmol/mol (6.5%) or higher are indicative of diabetes [2, 3]. Fasting and postprandial hyperglycemia, as well as relative insulin deficiency, are the defining characteristics of all forms of diabetes [4]. Retinopathy, nephropathy, and debilitating neuropathy are microvascular complications that can be caused by DM [5]. Diabetes is also a primary cause of macrovascular complications, which are typically related to cardiovascular diseases (CVDs) such as coronary artery disease, atherosclerosis, and hypertension [6]. Moreover, microvascular complications, such as diabetic nephropathy, are strongly linked to accelerated rates of CVD and atherosclerosis [7]. Several pathological factors, including hyperglycemia, hyperlipidemia, advanced glycation end products (AGEs), growth factors, and inflammatory cytokines/chemokines, have been associated with the increased incidence of these complications in the diabetic population [8, 9]. The development of diabetes is influenced by a variety of environmental factors, including obesity, smoking, and alcohol consumption [10]. Nonetheless, genetic factors have been identified as important moderators of individual susceptibility to Type 2 diabetes (T2D) development and response to lifestyle interventions [11]. These facts provide evidence for the necessity of further research into the molecular mechanisms of DM and the identification of novel pathogenesis-related factors [12]. In this article, we investigated the effects of astaxanthin (ASX) supplementation on microRNAs (miRNAs), lysophosphatidylcholine (LPC), and α-hydroxybutyrate (α-HB) as novel factors in the development of a variety of diabetes-related complications [13–15].

LPC is believed to play a significant role in atherosclerosis and inflammatory diseases by modifying the functions of multiple cell types, including smooth muscle cells, endothelial cells, monocytes, macrophages, and T cells [16]. LPC can interfere with glucose-stimulated insulin secretion (GSIS) by impairing calcium homeostasis and other signaling pathways that are crucial for the proper functioning of beta cells. Furthermore, this impairment exacerbates hyperglycemia in diabetic patients [15, 17]. LPCs may impede insulin signaling pathways, thereby contributing to insulin resistance (IR). Primarily, LPC has the ability to activate the protein kinase C (PKC) and mitogen-activated protein kinase (MAPK) pathways, which are recognized for their ability to impair insulin receptor substrate (IRS) function, thereby decreasing insulin sensitivity [15, 18–20]. α-HB is also an indicator of IR and impaired glucose regulation, both of which appear to result from excessive lipid oxidation and oxidative stress [21, 22]. The European population cohorts in 2016 identified α-HB as a selective biomarker for decreased glucose tolerance and prediabetes, which was independent of age, sex, BMI, and fasting glucose [13].

miRNAs are small regulatory RNAs, typically consisting of 17–21 nucleotides, that exert control over the translation and stability of mRNA molecules. This regulation occurs via improper base pairing, specifically within the 3′ untranslated region of mRNA targets [23, 24]. Several dysregulated miRNAs originating from insulin-sensitive tissues, such as skeletal muscle, white adipose tissue, and insulin-producing pancreatic β-cells, have been associated with several processes relevant to diabetes, including insulin production, pancreatic β-cell function, and adipocyte differentiation [25, 26]. Several studies indicate that miRNAs such as miR-21, miR-132, miR-34a, miR-38, and miR-155 play a significant role in the etiology and pathogenesis of DM and its complications [27, 28]. The development and pathology of T2D, as well as the homeostasis of glucose and lipid metabolism, are associated with an increasing number of miRNA profiles [29]. Recent evidence suggests that miRNAs might assist as new biomarkers of IR and adiposity, as well as for monitoring therapy response in terms of glycemic target and for diabetes complications [30, 31]. In addition, a number of studies have established a link between miR-21, miR-34a, and miR-155 and diabetic complications (DCs) such as retinopathy and nephropathy. In 2006, Taganov et al. determined that certain lipopolysaccharides (LPS), particularly the TLR4 ligand, affected miRNA expression in human THP-1 monocytes, with miR-155 being one of the three increased miRNAs [32]. Previous research indicates that miR-34a directly targets p53 and is essential for p53-mediated biological processes, including cell cycle arrest, apoptosis, and senescence [33].

ASX is a xanthophyll carotenoid with a chemical composition of 3,3′-dihydroxy-, β,β′-carotene-4,4′-dione and the formula C_40_H_52_O_4_ [34]. In the United States, Europe, and Japan, ASX is known as a dietary supplement. One of the few antioxidants that can circulate throughout the entire body, protect every cell, and prevent lipid oxidation is ASX, as demonstrated by laboratory and clinical investigations [35]. The plasma glucose and insulin levels of high-fat fructose diet (HFFD)-fed mice were substantially reduced and insulin sensitivity was enhanced by ASX (6 mg/kg per day for 45 days), as reported by Arunkumar et al. [36]. Additionally, Nishida et al. demonstrated that glucose incorporation into skeletal muscles in ASX-treated HFD-fed mice was enhanced, resulting in improved glucose metabolism [37]. Numerous transcription factors, hormones, cytokines, enzymes, receptors, and signaling proteins are among the many molecular targets of ASX [38].

From a clinical point of view, the fluctuation of miR-21, miR-34a, and miR-155 in the circulation can serve as an indicator of T2D progression [39]. Moreover, few studies have focused on the antioxidant and anti-inflammatory effects of ASX on miRNAs in diabetic subjects. ASX regulates the expression of miRNAs by modulating oxidative stress and inflammatory signaling pathways, including NF-κB, Nrf2, and PI3K/Akt. This influences the regulation of downstream target genes that are involved in cellular protection and homeostasis [40, 41].

Therefore, we aimed to examine the effects of ASX supplementation on the expression of inflammation-related miRNAs, including hsa-miR-21, hsa-miR-34a, and hsa-miR-155, in T2D patients.

## 2. Materials and Methods

### 2.1. Participants

The trial applied a double-blind, parallel-group, placebo-controlled design and was carried out at a Diabetes Center of Medical Sciences, Iran. Before commencing the study, all patients endured an initial screening evaluation consisting of a review of their medical history, a physical examination, measurement of their vital signs, electrocardiogram (ECG), and laboratory parameter monitoring. The American Diabetes Association (ADA) criteria were utilized to identify diabetes, FPG 126 mg/dL or greater, HbA1c 6.5% or greater, or OGTT 200 mg/dL for at least 3 months [42]. Subjects were enrolled in the study if they fulfilled the following inclusion criteria: patients aged 20–60 years old who only received oral metformin for diabetes treatment and a disease duration between 1 and 7 years. Exclusion criteria included the presence of kidney or liver failure, chronic inflammatory disease, pulmonary or CVD, thyroid disorders, cancer, pregnancy or lactation, smoking or alcohol consumption, or surgery within the previous 6 months. Appointments for outpatient follow-up were scheduled on the day the treatment was initiated and subsequently at weeks 4, 8, and 12. A comprehensive analysis of adverse events (AEs), including treatment-emergent events, instances of symptomatic hypoglycemia, and medical laboratory information, was performed to assess the drug's safety. Serum biochemistry analyses were performed to evaluate liver and kidney function.

### 2.2. Study Design

Volunteers with T2D were recruited for a randomized, double-blind, 3 month clinical trial. The study sample size was calculated based on the FPG variable, which represented one of the primary endpoint measurements. This finding was based on the results of our pilot study, which was performed in a comparable setting and included a comparable population of patients recently diagnosed with T2DM. Changes in the mean value of FPG were used to determine the sample size. To detect a 25% change in the primary outcome at a two-sided significance level of 0.05 and a power of 0.8, each group needed 25 participants. In addition, because we anticipated a 20% attrition rate, this study was designed to necessitate an ultimate sample size of 30 patients per group. The original intended sample size for the study was 30 participants per group, but it ended up with only 25 participants per group. The decline in participant numbers was primarily due to participant dropout, which was caused by their unwillingness to continue, the need for insulin treatment, and occasionally, surgical interventions. The primary outcome was a change in inflammation-related miRNA gene expression, α-HB, and LPC in peripheral blood mononuclear cells (PBMCs) from baseline to the end of the trial. Secondary outcomes were blood urea nitrogen (BUN), serum creatinine (Cr), uric acid, urinary albumin-to-creatinine ratio (ACR), lipid profile, FPG, and HbA1c levels. The subjects were requested to submit 3-day food records at the commencement, midpoint, and end of the study in order to evaluate their typical diets. These records were to include 2 days of regular eating and one day of rest. We analyzed and calculated the data from the dietary records using Nutritionist IV software (First Databank, San Bruno, CA).

Randomization was conducted utilizing a computer-generated random number sequence (RAS/Version 1.0.0 software) across 4 blocks to divide the 2 groups. The researchers and participants were blinded to the randomization and allocation of participants until the final data analysis. On the containers were the letters A, B, C, or D, with A and B representing placebos and C and D depicting ASX capsules. The patients and the researchers were blinded to the contents of the containers. We followed the statistical models of Mahdi Tavakolizadeh et al. [43] and Rad et al. [44], as well as the study model of Sharifi-Rigi et al. [45]. Also, the methodological framework of this study follows the approach detailed in the doctoral dissertation of the first author [45]. The Ethics Committee at Shiraz University of Medical Sciences approved the study protocol with Ethics Code IR.SUMS.REC.1401.029, and was conducted by the Declaration of Helsinki. Participants were informed about the study's objectives and provided written informed consent. Confidentiality and anonymity were maintained throughout the study.

### 2.3. Intervention and Follow-Up

All patients were assigned randomly to one of two research groups (each containing 25 patients). Group 1 (the control group) was administered placebo capsules (Barij Essence Pharmaceutical Company, Iran) once per day for a period of 12 weeks. To ensure blinding integrity, the placebo capsules matched the ASX capsules in color, size, and shape. The inactive substances in the supplement and placebo were dicalcium phosphate, microcrystalline cellulose, stearic acid, silicon dioxide, and magnesium stearate. Group 2 was administered 10 mg of ASX capsules (produced by haematococcus pluvialis microalgae; LOT No: 8277, Waka Tani Health Nutrition Company, USA) once daily for 12 weeks [45, 46]. Then, in the ASX group, the participants were divided into 2 subgroups according to the ACR index (ACR < 30 mg/g or ACR ≥ 30 mg/g). During the clinical trial, all patients were instructed not to alter their oral hypoglycemic medications, normal diet, or daily physical activities or to take any supplements. The participants were followed up on a weekly basis via telephone interviews. Compliance with supplements was evaluated by the number of unused capsules remaining at the end of each week.

### 2.4. Blood Samples and Isolation of PBMCs

The night prior to urine and blood collection, subjects were instructed to fast for 12 h. Blood and urine samples were collected at the beginning and end of the study. Blood samples were taken between 8 and 10 a.m. to minimize circadian variations. All serum and plasma samples were frozen at −80°C until analysis. Using 12 mL blood samples collected in heparinized tubes (Vacutainer, Franklin Lakes, NJ, USA), PBMCs were isolated. PBMCs for the real-time PCR assay were separated by density-gradient centrifugation with Ficoll–Hypaque (Cedarlane Laboratories, Hornby, ON, Canada) [47].

### 2.5. Anthropometric Measurements

A standard scale (Seca, Hamburg, Germany) was employed to determine the individual's weight and height at the start and finish of the therapy. To calculate BMI, the weight in kg was divided by the square of the height in meters.

### 2.6. Biochemical Analysis

FPG, BUN, Cr, uric acid, and lipid profiles were measured using an autoanalyzer instrument (BT300, Rome, Italy) and a commercially available kit (Pars Azmoon Co., Tehran, Iran). HbA1c in the whole blood sample was determined using DS5 chromatography (Drew Scientific Inc., France). ACR was determined as an indicator of diabetic kidney disease (DKD) [48, 49]. The HOMA-IR index was calculated using the equation proposed by Matthews et al.: plasma glucose (mmol/L) × insulin (μU/L)/22.5 [50].

### 2.7. ELISA Analysis

LPC (Cat No: ab273332, Abcam), α-HB (Cat No: CELI-66002h, Gentaur), and inflammatory cytokines, including TNF-α (Cat No: E0082Hu), IL-6 (Cat No: E3764Hu), IL-1β (Cat No: E0143Hu) serum levels, were assessed using enzyme-linked immunosorbent assay (ELISA) reagents in accordance with the manufacturer's guidance.

### 2.8. RNA Extraction, cDNA Synthesis, and qRT‐PCR Analysis

According to the manufacturer's instructions, total RNA was extracted from peripheral blood samples using RNXTM-PLUS solution (CinnaGen, Tehran, Iran). The purity and concentration of RNA were then determined using a NanoDrop (Thermo Fisher Scientific, USA) spectrophotometer by computing the absorbance ratio at 260/280 nm. The process of cDNA synthesis was conducted using 1 μg of total RNA and 1 pM of stem-loop-specific primers for miRNAs and housekeeping genes. Reverse transcription reactions were conducted at 37°C for 60 min in accordance with the manufacturer's instructions (BiomiR high-sensitivity miRNA kit, Anacell, Tehran, Iran). qRT-PCR was applied to quantify the relative expression levels of hsa-miR-21, hsa-miR-34a, and hsa-miR-155. The qRT-PCRs were conducted in duplicate for each target miRNA. Each reaction consisted of 2 μL of cDNA, 10 μM of specific primers, and 2xQPCR Master Mix SYBR Green I. The RT–PCR program included a single cycle at 95°C for 15 min, followed by 40 two-step cycles at 95°C for 15 s and 55°C–60°C for 60 s. The RT-PCR program included a single cycle at 95°C for 15 min, followed by 40 two-step cycles at 95°C for 15 s and 55°C–60°C for 60 s (Anacell, Tehran, Iran). U6 snRNA was utilized as a candidate endogenous control miRNA for data normalization. The reactions were performed on a LightCycler instrument (LightCycler 96, Roche, Germany), and the results were subsequently analyzed using the 2^−ΔCT^ and 2^−ΔΔct^ procedures outlined by Livak and Schmittgen [51].

### 2.9. Statistical Analysis

Data are displayed as the mean ± standard deviation. The Kolmogorov-Smirnov test was used to assess the normality of the data. A paired *t*-test was used to analyze variable changes within-group. We conducted an independent *t*-test for parametric distributions or a Mann–Whitney test for nonparametric distributions to compare the quantitative continuous variables between groups. After adjusting the baseline values of both groups, analysis of covariance (ANCOVA) was performed to compare post-treatment variables. The mean changes of the outcome variables between the groups were compared using the ANCOVA test, which was conducted after adjusting for baseline values, age, sex, and baseline BMI, in order to control for confounders. The reference gene was U6, which was also used as a control gene, and the (2^−ΔCT^) formula and fold change were used to calculate the relative expression of the gene in PBMCs. Pearson correlation was applied to check out intervariable relationships. While the intention-to-treat (ITT) approach was introduced, the final statistical analyses were performed on the per-protocol (PP) population, consisting of participants who completed the study according to the protocol. This involved the inclusion and analysis of all randomly assigned participants according to their original group. Using receiver-operating characteristic (ROC) curve analysis, the potential of each miRNA to discriminate between groups of study subjects was evaluated, and the optimal threshold was determined using Youden's method. Statistical analyses were performed using SPSS Version 23.0 (IBM, Armonk, NY, USA). *p* < 0.05 was considered statistically significant. Figures were drawn using Version 6.0 of Prism software (GraphPad, California, United States).

## 3. Results

### 3.1. Flowchart of the Study Design and Protocol

**Figure 1:**
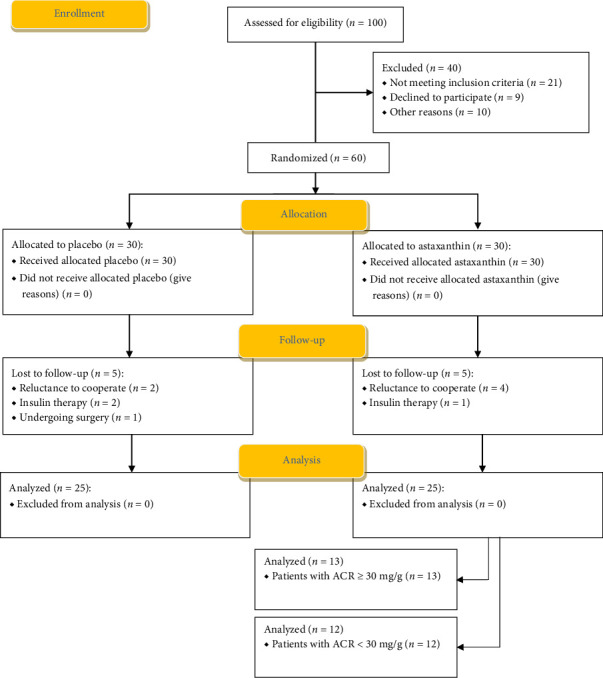
Flowchart of the study design and protocol.

The trial design and flowchart are depicted in Figure 1. A total of 100 patients were screened for eligibility, as depicted in the flowchart. Of these, 40 patients were excluded (not meeting eligibility requirements [*n* = 21], refusing participation [*n* = 9], and for other reasons [*n* = 10]), and ultimately, 60 diabetic volunteers were randomly assigned to the ASX or placebo groups for the 12-week run-in phase trial. Ten patients dropped out of the study after randomization because they were transferred to insulin therapy (*n* = 3), were reluctant to cooperate (*n* = 6), or underwent surgery (*n* = 1). Consequently, 50 patients were incorporated into the statistical analysis of compliance with therapy (25 participants in each group).

### 3.2. Baseline Characteristics and Biochemical Analysis Results

#### 3.2.1. Baseline Demographic Characteristics and Clinical Parameters of the Study Population

Supporting Information Table 1 (see Supporting Information) presents the baseline demographic characteristics of the two groups of participants. Supporting Information Table 1 details the percentages of males and females, the mean age, duration of diabetes, body weight, height, BMI, and clinical parameters. There were no significant differences in demographic characteristics between the two groups at baseline (*p* > 0.05).

#### 3.2.2. Effects of ASX on Biochemical Parameters

Supporting Information Table 2 (see Supporting Information) displays the effects of ASX on biochemical parameters. The level of FPG before and after 12 weeks of treatment with ASX was 139.27 ± 21.18 vs. 126.43 ± 18.97 (*p*=0.002), demonstrating a significant reduction compared to the placebo group. In the placebo group, the changes in glycemic parameters after 12 weeks of treatment were not statistically significant (Supporting Information Table 2). In the ASX group, the mean HbA1c level at baseline was 7.89 ± 0.79 and declined to 7.05 ± 0.35 after the supplementation period, which was statistically significant (*p* < 0.001) (Supporting Information Table 2). In addition, supplementation with ASX resulted in a statistically significant drop in HOMA-IR levels (*p* < 0.001), whereas this parameter was not altered significantly in the placebo group (Supporting Information Table 2). In the ASX group, at baseline, serum total cholesterol, TG, and LDL were 150.98 ± 39.25, 142.37 ± 52.71, and 91.36 ± 28.36, respectively. Daily consumption of ASX capsules significantly reduced total cholesterol, TG, and LDL to 130.65 ± 36.15, 131.25 ± 39.81, and 69.05 ± 36.98, respectively. The ASX group, in comparison with the placebo group, demonstrated marked changes in lipid profile factors such as TC, TG, and LDL (*p*=0.011, *p*=0.043, and *p*=0.022, respectively) (Supporting Information Table 2).

#### 3.2.3. Effects of ASX on Renal Indicators in Patients With ACR ≥ 30 mg/g in the ASX Subgroup

As indicated in Table 1, the baseline urinary ACR, serum urea, creatinine, and uric acid for the participants with ACR ≥ 30 mg/g in the ASX subgroup were 34.46 ± 7.36, 37.63 ± 9.72, 1.3 ± 0.05, and 5.71 ± 1.48, respectively. Consuming ASX capsules on a daily basis substantially reduced (*p* < 0.05) urinary ACR (*p*=0.003), BUN (*p*=0.006), Cr (*p*=0.009), and uric acid (*p*=0.014) to 25.39 ± 8.22, 28.01 ± 8.61, 8.4 ± 0.07, and 4.37 ± 1.93, respectively.

### 3.3. qRT‐PCR Analysis Results

#### 3.3.1. Differences in Inflammation-Related miRNA Expression in Patients With ACR ≥ 30 or < 30 mg/g in ASX Subgroups

In Table 2, the differences in inflammation-related miRNA fold change expression between the subjects with and without albuminuria are shown in ASX subgroups. We observed that the group of patients with albuminuria (ACR ≥ 30 mg/g) had significantly higher (*p* < 0.05) hsa-miR-21 (*p*=0.010), hsa-miR-34a (*p*=0.008), and hsa-miR-155 (*p*=0.004) expression than the group of patients without albuminuria (ACR < 30 mg/g). Furthermore, Supporting Information Table 3 displays the −ΔCt values for each miRNA, which suggest that the expression levels are elevated in patients with ACR ≥ 30 mg/g. These findings, which are consistent with the fold change analysis, further confirm the upregulation of these miRNAs in this patient group.

#### 3.3.2. Effects of ASX on Inflammation-Related miRNA Expression in PBMCs

**Figure 2:**
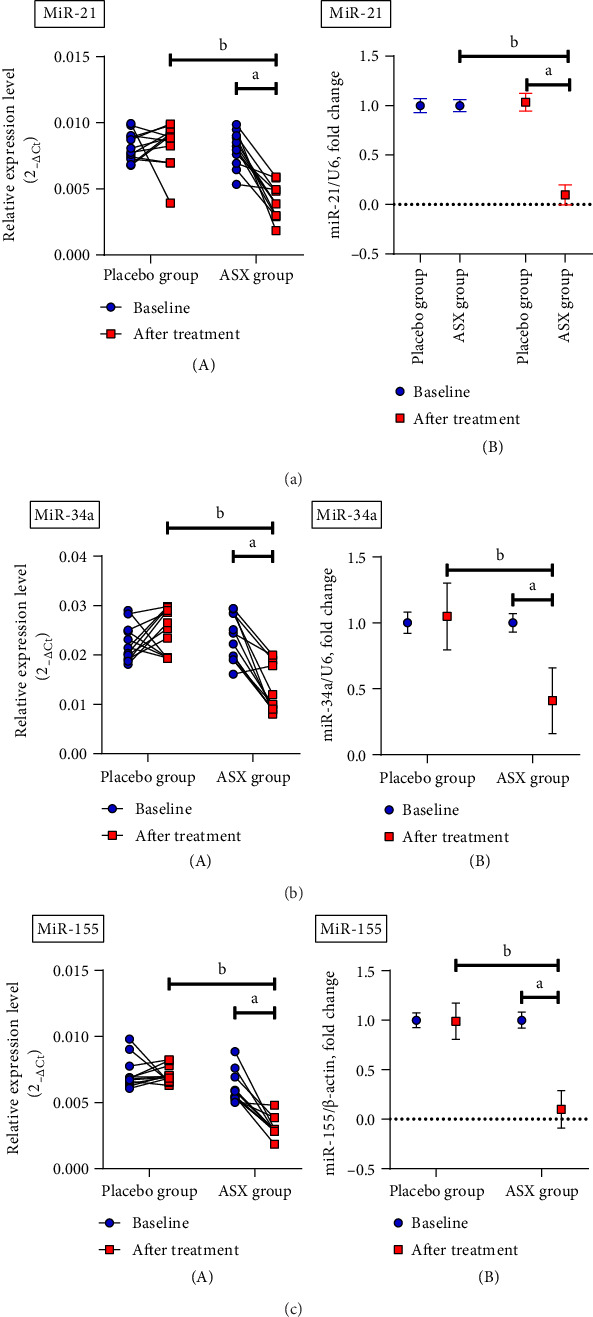
Evaluation of the 12-week supplementation with 10 mg/day ASX or placebo on hsa-miRNAs gene expression in PBMCs of T2D patients. The data are expressed as the mean ± SD, and *n* = 25 biological replicates in each group. ^a^*p* < 0.05 by paired sample *t*-test shows a significant difference within the astaxanthin group after vs. before supplementation. ^b^*p* < 0.05 by independent samples' *t*-test indicates a significant difference between astaxanthin supplementation and placebo. Abbreviations: ASX = astaxanthin; hsa-miRNAs = *Homo sapiens* microRNAs; miR-21 = microRNA-21; miR-34a = microRNA-34a; miR-155 = microRNA-155; PBMCs = peripheral blood mononuclear cells; T2D = type 2 diabetes; SD = standard deviation.

**Figure 3:**
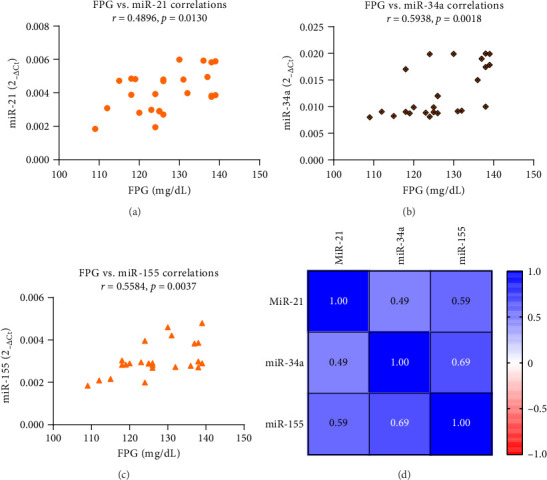
Correlation analyses of miRNAs with FPG (a–c) in T2D patients after 12 weeks of ASX supplementation (*n* = 25 biological replicates). Pearson correlation was used. Data are presented as 2^−ΔCt^ levels for miRNAs and mg/dL for FPG; (d) correlation between miRNAs, and the color shift in the heatmap shows that red indicates the indirect association, and blue indicates the direct link between miRNAs. Abbreviations: ASX = astaxanthin; FPG = fasting plasma glucose; miR-21 = microRNA-21; miR-34a = microRNA-34a; miR-155 = microRNA-155; T2D = Type 2 diabetes.

**Figure 4:**
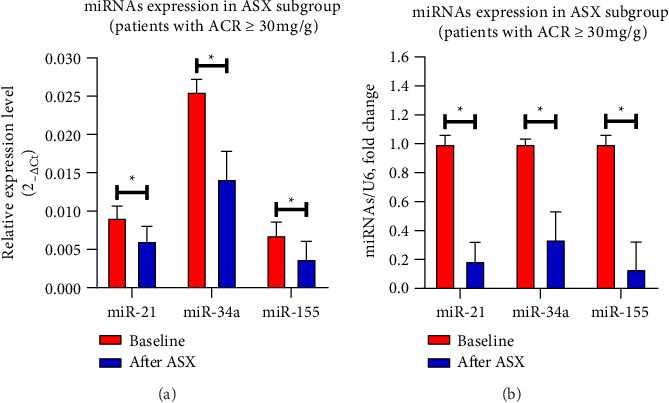
Evaluation of the 12-week supplementation with 10 mg/day ASX on inflammation-related miRNA gene expression in PBMCs of T2D patients with DKD (*n* = 13 biological replicates). The data are expressed as the mean ± SD. ⁣^∗^*p* < 0.05 by paired sample *t*-test shows a significant difference within the astaxanthin subgroup after vs. before supplementation. Abbreviations: ACR = albumin-to-creatinine ratio; ASX = astaxanthin; DKD = diabetic kidney disease; miR-21 = microRNA-21; miR-34a = microRNA-34a; miR-155 = microRNA-155; PBMCs = peripheral blood mononuclear cells; SD = standard deviation; T2D = Type 2 diabetes.

**Figure 5:**
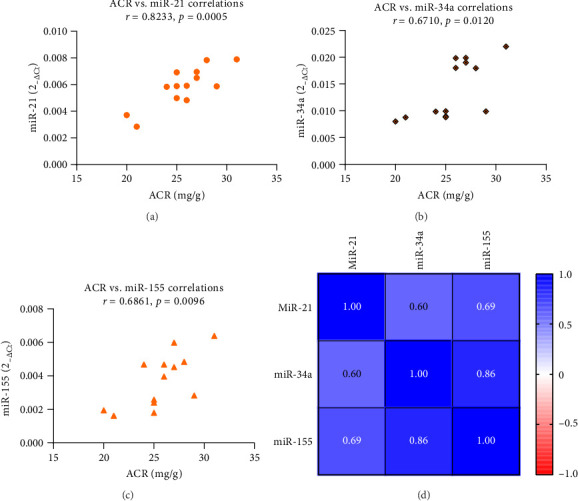
Correlation analyses of miRNAs with ACR (a–c) in T2D patients with DKD after 12 weeks of ASX supplementation (*n* = 13 biological replicates). Pearson correlation was used. Data are presented as 2^−ΔCt^ levels for miRNAs and mg/g for ACR; (d) correlation between miRNAs, and the color shift in the heatmap shows that red indicates the indirect association and blue indicates the direct link between miRNAs. Abbreviations: ACR = albumin-to-creatinine ratio; ASX = astaxanthin; miR-21 = microRNA-21; miR-34a = microRNA-34a; miR-155 = microRNA-155; T2D, Type 2 diabetes.

Figure 2 shows the effects of ASX on miR-21, miR-34a, and miR-155 gene expression in PBMCs from the experimental groups. ASX led to a considerable decrease (*p* < 0.05) in hsa-miR-21 (*p* < 0.001), hsa-miR-34a (*p* < 0.001), and hsa-miR-155 (*p* < 0.001) gene expression compared with the placebo. Within the ASX group, the reduction in hsa-miR-21 (*p* < 0.001), hsa-miR-34a (*p* < 0.001), and hsa-miR-155 (*p* < 0.001) gene expression was also statistically significant (*p* < 0.05) after supplementation vs. baseline. The expression levels of inflammation-related miRNAs such as hsa-miR-21 (*R*^2^ = 0.2397; 95% CI = 0.1171 − 0.7413; *p*=0.0130) (Figure 3(a)), hsa-miR-34a (*R*^2^ = 0.3526; 95% CI = 0.2595 − 0.8010; *p*=0.0018) (Figure 3(b)), and hsa-miR-155 (*R*^2^ = 0.3118; 95% CI = 0.2095 − 0.7812; *p*=0.0037) (Figure 3(c)) were significantly positively correlated with the level of FPG (*p* < 0.05). Figure 3(d) depicts a miRNA association heatmap in the ASX group. Moreover, significant positive correlations were observed between inflammation-related miRNAs and the proinflammatory cytokines TNF-α (*p* < 0.05; Supporting Information Figure 1-1), IL-6 (*p* < 0.05; Supporting Information Figures 1 and 2), and IL-1β (*p* < 0.05; Supporting Information Figure 1-3). Additionally, in the participants with ACR ≥ 30 mg/g in the ASX subgroup, a comparison of inflammation-related miRNA gene expression at 12 weeks of supplementation vs. baseline revealed a significant reduction in hsa-miR-21 (*p* < 0.001), hsa-miR-34a (*p* < 0.001), and hsa-miR-155 (*p* < 0.001) (Figure 4). The correlation analysis revealed positive correlations between inflammation-related miRNAs, including hsa-miR-21 (*R*^2^ = 0.6778; 95% CI = 0.4984 − 0.9454; *p*=0.0005) (Figure 5(a)), hsa-miR-34a (*R*^2^ = 0.4503; 95% CI = 0.1905 − 0.8922; *p*=0.0120) (Figure 5(b)), and hsa-miR-155 (*R*^2^ = 0.4707; 95% CI = 0.2173 − 0.8977; *p*=0.0096) (Figure 5(c)) expression and ACR. Figure 5(d) shows a miRNA positive correlation (blue) heatmap for the participants with ACR ≥ 30 mg/g in the ASX subgroup.

### 3.4. ELISA analysis Results

#### 3.4.1. Effects of ASX on Serum Levels of LPC, α-HB, and Proinflammatory Cytokines

**Figure 6:**
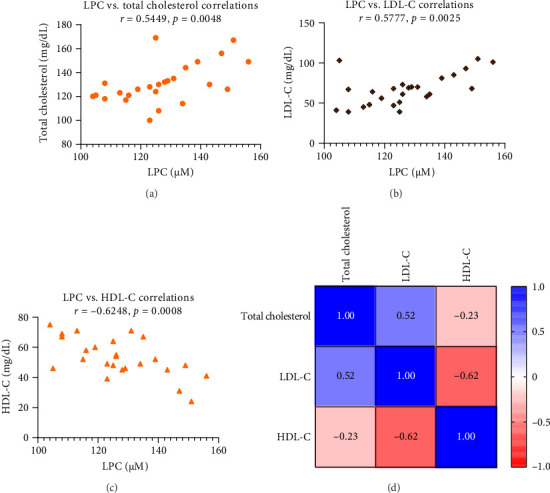
Correlation analyses of total cholesterol (a), LDL-C (b), and HDL-C (c) with LPC in T2D patients after 12 weeks of ASX supplementation (*n* = 25 biological replicates). Pearson correlation was used. Data are presented as mg/dL levels for total cholesterol, LDL-C, and HDL-C and μM for LPC; (d) correlation between total cholesterol, LDL-C, and HDL-C, and the color shift in the heatmap shows that red indicates the indirect association and blue indicates the direct link between total cholesterol, LDL-C, and HDL-C. Abbreviations: ASX = astaxanthin; HDL-C = high-density lipoprotein cholesterol; LDL-C = low-density lipoprotein cholesterol; LPC = lysophosphatidylcholine; T2D = type 2 diabetes.

**Figure 7:**
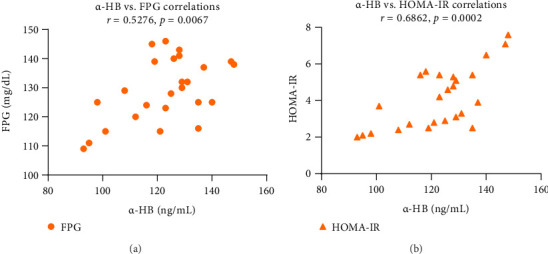
Correlation analyses of FPG (a) and HOMA-IR (b) with α-HB in T2D patients after 12 weeks of ASX supplementation (*n* = 25 biological replicates). Pearson correlation was used. Data are presented as mg/dL levels for FPG and ng/mL for α-HB. Abbreviations: α-HB = alpha-hydroxybutyrate; ASX = astaxanthin; FPG = fasting plasma glucose; HOMA-IR = homeostatic model assessment for insulin resistance; T2D = Type 2 diabetes.

Table 3 demonstrates the effects of ASX on the serum levels of LPC and α-HB. In the ASX group, LPC and α-HB serum levels decreased significantly (*p* < 0.05) after supplementation with ASX, whereas there was no statistically significant change in these parameters in the placebo group (Table 3). Serum levels of LPC were positively correlated with total cholesterol (*R*^2^ = 0.2970; 95% CI = 0.1909 − 0.7735; *p*=0.0048) (Figure 6(a)) and LDL-C (*R*^2^ = 0.3337; 95% CI = 0.2365 − 0.7920; *p*=0.0025) (Figure 6(b)). Nevertheless, there was a significant negative correlation between serum LPC and HDL-C (*R*^2^ = 0.3904; 95% CI = −0.8180 − −0.3050; *p*=0.0008) (Figure 6(c)), which may indicate the relevance of LPC in diabetes-related atherosclerotic complications. Additionally, Figure 6(d) shows the total cholesterol, LDL-C, and HDL-C association heatmap in the ASX group. Furthermore, the serum level of α-HB showed a significant positive correlation with the levels of FPG (*R*^2^ = 0.2784; 95% CI = 0.1674 − 0.7636; *p*=0.0067) (Figure 7(a)) and HOMA-IR (*R*^2^ = 0.4709; 95% CI = 0.3993 − 0.8507; *p*=0.0002) (Figure 7(b)), suggesting a contributory role for α-HB in IR. Supporting Information Table 4 displays the impact of ASX on the serum levels of proinflammatory cytokines. Following ASX supplementation, the serum levels of TNF-α (*p*=0.008), IL-6 (*p*=0.004), and IL-1β (*p*=0.003) decreased significantly in the ASX group, whereas the placebo group did not experience a statistically significant change in these parameters.

### 3.5. ROC Analysis Results

#### 3.5.1. ROC Analysis of hsa-miR-21, hsa-miR-34a, and hsa-miR-155 Expression in T2D Patients With ACR ≥ 30 mg/g

**Figure 8:**
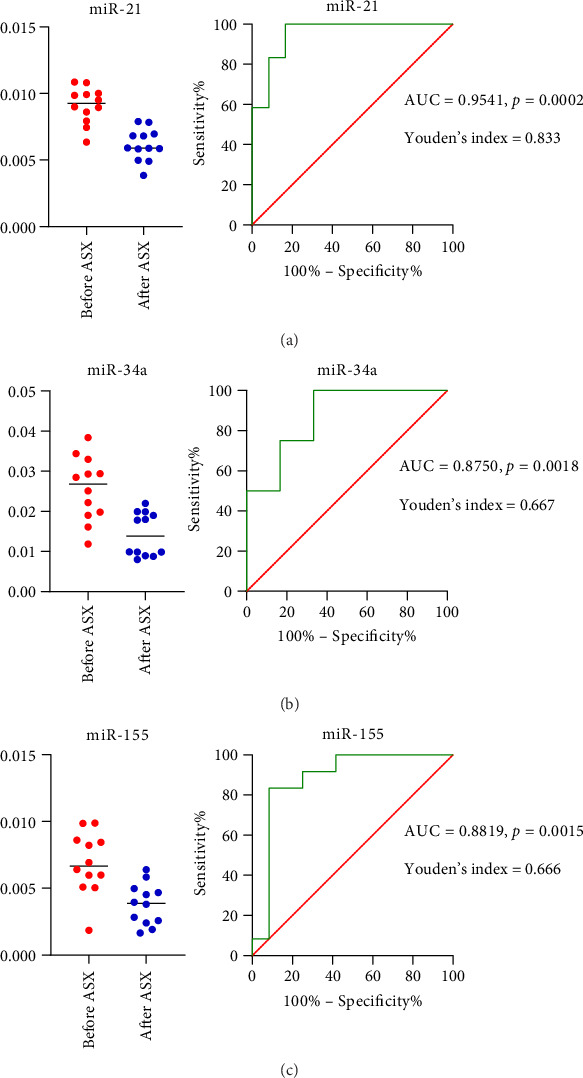
ROC curve of inflammation-related miRNA expression in the context of DKD. Abbreviations: ASX = astaxanthin; AUC = area under curve; DKD = diabetic kidney disease; miR-21 = microRNA-21; miR-34a = microRNA-34a; miR-155 = microRNA-155; ROC = receiver operating characteristic.

ROC curve analysis was conducted to explore the potential of inflammation-related miRNAs as supportive markers for DKD (ACR ≥ 30 mg/g) in patients with T2D. The area under the curve (AUC) for miR-21, miR-34a, and miR-155 were 0.9541 (95% CI = 0.8699 − 0.975; *p* < 0.001) (Figure 8(a)), 0.8750 (95% CI = 0.7364 − 0.934; *p* < 0.001) (Figure 8(b)), and 0.8819 (95% CI = 0.7266 − 0.928; *p* < 0.001) (Figure 8(c)), respectively. At a cutoff value of 0.0098 for miR-21, the sensitivity was 91.67%, and the specificity was 83.33%. At the cutoff value of 0.02209 for miR-34a, the sensitivity was 91.33%, and the specificity was 66.67%. At a cutoff value of 0.005910 for miR-155, the sensitivity was 85.67%, and the specificity was 83.33%, demonstrating that miR-21, miR-34a, and miR-155 expression may serve as adjunctive indicators for T2D with DKD (Figure 8).

#### 3.5.2. ROC Analysis of LPC in Relation to Atherosclerotic Risk

**Figure 9:**
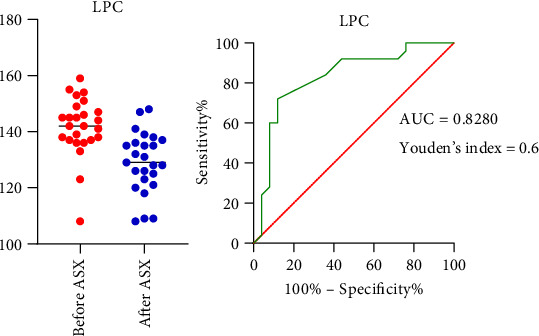
ROC curve of serum LPC in relation to atherosclerotic risk. Abbreviations: ASX = astaxanthin; AUC = area under curve; LPC = lysophosphatidylcholine; ROC = receiver operating characteristic.

ROC curve analysis was applied to assess the potential diagnostic value of LPC as a supportive indicator related to atherosclerotic risk. The AUC was 0.8280 (95% CI = 0.7077 − 0.9483; *p* < 0.001), with a sensitivity of 92% and a specificity of 56% (Figure 9), suggesting that plasma LPC levels may have shown potential utility as exploratory biomarkers for atherosclerotic complications.

#### 3.5.3. ROC Analysis of α-HB in Relation to IR

**Figure 10:**
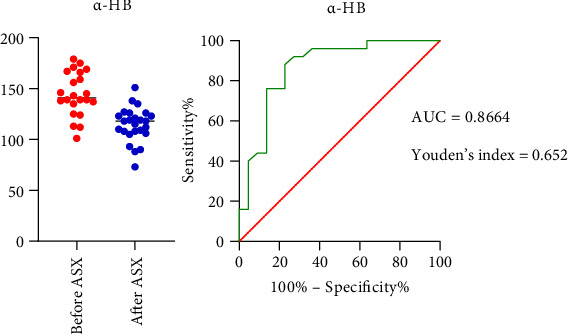
ROC curve of serum α-HB in relation to IR. Abbreviations: α-HB = alpha-hydroxybutyrate; ASX = astaxanthin; AUC = area under curve; IR = insulin resistance; ROC = receiver operating characteristic.

ROC curve analysis was used to explore the potential of using α-HB as a biomarker for IR. The AUC was 0.8664 (95% CI = 0.7571 − 0.9756; *p* < 0.001), with a sensitivity of 96% and a specificity of 64% (Figure 10). The results indicated that the α-HB levels were potentially associated with IR.

## 4. Discussion

Type 2 diabetes is linked to oxidative stress and low-grade inflammation, which result in DCs. Moreover, elevated levels of oxidative stress and inflammation have the potential to induce IR and impair the release of insulin [52, 53]. Changes in epigenetic regulation may likewise impact these processes. Clinical studies indicate that rigorous diabetes management does not substantially diminish the appearance of DC [54]. Modifications in oxidative stress and IR markers, as well as miRNA expression, must be analyzed to identify biological markers with sufficient predictive power for the development of complications in diabetic patients [55]. Subsequently, using a safe supplement with potent antioxidant properties can prevent DCs resulting from inflammation [56].

In this randomized, double-blind, and placebo-controlled study, adjusting for baseline differences using ANCOVA, we assessed the effects of 10 mg/day ASX supplementation for 12 weeks on α-HB, LPC, and inflammation-related miRNAs in individuals with T2D. We found that supplementation with ASX substantially diminished the levels of α-HB, LPC, and inflammation-related miRNAs in diabetic patients with and without complications.

In the present investigation, we utilized PBMCs to investigate miRNAs involved in inflammation. Since accessibility to tissue is a limitation in human studies, however, surveillance of gene expression in PBMCs may expose the metabolic and immunological reactions of adipose tissue, pancreatic β-cells, and hepatic tissue [57]. Additionally, the capacity of PBMCs to precisely represent the impact of dietary interventions on gene expression has demonstrated their viability as a viable organ for nutrigenomics research [58, 59]. In prior studies, the gene expression of PBMCs following dietary intervention was used to investigate the effect of supplementation treatments on miRNAs related to T2D [60, 61].

There is mounting evidence that miRNAs and metabolites play a role in the pathogenesis of diseases. As a consequence, a number of miRNAs, such as miR-21 [62], miR-34a [63], and miR-155 [64], in addition to metabolites, such as LPC [16] and α-HB [65], have been proposed as potential novel biomarkers for IR and DC.

Recent research suggests that miRNAs, which regulate the expression of critical genes, may play a role in the pathogenesis of a wide variety of human diseases [66]. On the other hand, miRNAs have garnered considerable attention as a result of their regulatory effects on gene expression. Fascinatingly, exosomes can be encapsulated in miRNAs to facilitate their transportation or distribution to the target cells or tissues, where they exert a physiological regulatory effect. As a result, exosomal miRNAs are demonstrating their significance as regulators of DM during its establishment and maintenance [67]. Additionally, the bioinformatics analysis of the preceding study identified markers that may be associated with the development, occurrence, and management of T2DM. The previous study also identified miRNAs, including hsa-mir-155-5p, hsa-miR-192-5p, hsa-miR-124-5p, hsa-miR-335-5p, hsa-mir-34a-5p, and miR-7110, as well as TFs, including Smad5 and Bcl6, that may be beneficial for the diagnosis and management of T2DM [68–70]. In silico investigations have previously identified tissue-specific differentially expressed genes (DEGs) in T2DM, predominantly in the heart, liver, and pancreas. The following hub miRNAs were identified as commonly targeted: hsa-let-7b-5p, hsa-miR-21-5p, hsa-miR-124-3p, hsa-miR-1-3p, and hsa-miR-155-5p. A variety of pathways, such as the endocrine resistance, phosphatidyl 3-kinase-protein kinase B, and AGE-RAGE signaling pathways, are regulated by these identified miRNAs [69, 71].

According to previous studies, inflammation-related miRNAs such as miR-21, miR-34a, and miR-155 exhibit multifunctional roles and are critically involved in various physiological and pathological processes, including immune regulation, inflammation, carcinogenesis, and cardiovascular disorders [27, 30, 55, 63, 72, 73]. In this research, we found significantly increased inflammation-related miRNA expression in diabetes patients with DKD (ACR ≥ 30 mg/g) compared with subjects with ACR < 30 mg/g (Table 2). Our data show a positive association between ACR and miRNAs (Figure 5), which implies the possible involvement of these miRNAs in DKD. Our ROC analysis demonstrated statistically significant associations between inflammation-related miRNAs and albuminuria (Figure 8). These findings suggest that such miRNAs may have a contributory role in the prediction of DKD; however, they should not be considered as independent diagnostic markers at this stage. Moreover, based on our findings (Supporting Information Figure 1), the observed positive correlations between inflammatory cytokines and miRNAs 21, 34a, and 155 may provide supporting evidence for a potential link between these miRNAs and inflammatory processes.

These miRNAs displayed pathogenic roles in DKD by creating an intricate network with targeted genes such as FOXO1, MMP-9, Smad7, TIMP3, Cdk6, and IMP3 and signaling cascades, including PKC, TGF-β/NF-κB/SMAD, Akt/TORC1, CADM1/STAT3, and the AGE-RAGE regulatory cascade, resulting in extracellular matrix deposition, cytoskeletal remodeling, epithelial-to-mesenchymal transition, inflammation, and fibrosis [74–76]. Also, miR-155 may be implicated in the early phases of diabetic nephropathy. Numerous studies have exhibited a substantial increase in miR-155 expression in glomerular mesangial cells (GMCs) in both human and rat species that are exposed to elevated glucose levels. Interestingly, the upregulation of miR-155 is reduced in TLR4-deficient cells; this implies that inflammation is a primary determinant in the transcriptional regulation of miR-155. The proinflammatory cytokines' induced increase in miR-155 expression in human GMCs supports this viewpoint [77]. On the other hand, studies have demonstrated that hyperglycemia increases inflammation-related miRNA expression through epigenetic alterations such as DNA methylation and histone modifications [78]. Our findings revealed a positive correlation between inflammation-related miRNAs and plasma glucose in diabetic patients (Figure 3). In this research, administration of ASX supplementation led to a significant decrease in hsa-miR-21, hsa-miR-34a, and hsa-miR-155 expression in T2D patients with and without DKD (Figure 2). Also, our results indicate that ASX supplementation can significantly reduce the levels of proinflammatory cytokines, including TNF-α, IL-6, and IL-1β, suggesting its potential anti-inflammatory properties. The obtained findings may be attributable to the effects of ASX on the expression of stress-responsive pathways, such as the Nrf-2/PGC-1α signaling axis, that regulate stress conditions [79]. Following supplementation, ASX may act as a catalyst for the upregulation of mitochondrial signaling proteins, including NAD-dependent deacetylases (SIRT1, SIRT3). This sirtuin family is recognized for its ability to enhance the activity of a variety of endogenous antioxidants, including heme oxygenase 1 (HO-1), catalases (CAT), and superoxide dismutase (SOD), by activating FOXO1, FOXO3, and Nrf-2 [80]. According to prior research, ASX ameliorated inflammation and IR by boosting the expression of PGC-1α through the AMPK/Sirt1 pathway [46]. Also, ASX has been demonstrated to possess antioxidant and anti-inflammatory properties through the reduction of PKC/NF-кB through both Nrf2-dependent and Nrf2-independent pathways in both in vivo and in vitro studies [81]. To verify the aforementioned, we can consult a study that examined the impact of ASX on the enhancement of mitochondrial adaptation during endurance training. In the context of mitochondrial regulation, this study implies that a mutually beneficial impact between ASX and endurance training may exist. Studies have shown that treating with ASX can fix issues with gene expression and protein modification in the mitochondria, while also making the metabolism more flexible and reducing oxidative damage in the body [80].

Phospholipase A2 (PLA2) catalyzes the conversion of phosphatidylcholines in LDL into LPC as a result of oxidative stress [82]. Recently, researchers have examined the significance of LPC in the development of endothelial disorders and arteriosclerosis, as LPC may be identified in high concentrations in arteriosclerotic plaques, with higher concentrations in symptomatic plaques relative to benign plaques [16].

As represented in our results, there is a significant correlation between LPC and atherosclerotic risk factors such as total cholesterol, LDL-C, and HDL-C (Figure 6), indicating the role of LPC in lipid oxidation. Furthermore, the ROC analysis revealed a statistical association between LPC levels and markers of atherosclerotic risk (optimal cutoff value ∼141.5 μM) (Figure 9). These findings suggest that LPC may serve as a supportive biomarker in the identification of cardiovascular complications in diabetes; however, its clinical utility remains to be established and requires further validation in larger, independent cohorts.

Through the combination of TLR2 and TLR4 receptors, LPC can stimulate the NF-kB, JUN, and p38 MAPK signaling pathways [16]. The activation of these processes may lead to the generation of proinflammatory factors, regulating inflammatory and infectious diseases. LPC can also trigger homocysteine-induced IR. An elevated level of LPC is associated with diabetic retinopathy and neurodegeneration as complications of diabetes [83, 84]. Oxidative stress conditions, such as diabetes, have long been acknowledged as a risk factor to CVDs. The inhibition of oxidative stress by dietary antioxidants, including vitamin E, carotenoids, and polyphenols, has been postulated in preclinical and epidemiological studies, offering cardiovascular protection [85]. The results of our study indicate that the serum level of LPC is considerably reduced in the ASX-treated group compared to the placebo group (Table 3), which is indicative of an improvement in lipid oxidation in groups supplemented with ASX as a result of its antioxidant properties [46]. ASX's antioxidant properties suggest that it has the potential to improve a diverse array of disorders that are affected by oxidative stress. Previously, a clinical trial has demonstrated that the administration of ASX may be a promising adjunct to address the inflammatory response and oxidative stress associated with endometriosis. Additionally, this pharmaceutical agent may enhance the quality of oocytes and embryos in endometriosis patients who attend ART clinics [86]. ASX has the potential to improve cognitive function, facilitate neuroprotection, and reduce neurodegeneration, according to another study. The established positive effects of ASX on a variety of memory and response time branches, as well as the implications of research conducted on biomarkers in human populations, are the foundation of this claim [87].

Lactate dehydrogenase (LDH) converts α-ketobutyrate (α-KB) into α-HB, an organic acid necessary for the metabolism of threonine and methionine–cysteine and the production of glutathione [65]. To discover metabolite biomarkers for the diagnosis of cancer, elevated α-HB levels have been detected in a variety of cancers, including hepatocellular carcinoma, colorectal cancer (CRC), gastric cancer, lung cancer, and B-cell lymphoma [88]. Moreover, elevated α-HB levels in the plasma of patients with diabetes predict IR and impaired glucose homeostasis, which is consistent with the upregulation of cysteine levels [89]. In our clinical trials, we found a significant positive correlation between the level of α-HB and HOMA-IR (Figure 7), indicating the role of these α-HBs in IR. Moreover, The ROC analysis results suggest that α-HB may have a contributory role in the relative identification of IR (optimal cutoff value ∼136 ng/mL) (Figure 10), particularly given its observed positive association with FPG and HOMA-IR levels. However, its interpretation should remain supportive and not replace established markers of IR.

The accumulation of α-HB may be the result of enhanced glutathione synthesis or the nuclear translocation of LDHA resulting from oxidative stress, including ROS and elevated NADH/NAD+ ratios, both of which are frequently involved in the development of diabetes and cancer [88]. According to research, ROS scavengers are able to substantially decrease LDHA nuclear translocation, suggesting that anti-inflammation via ROS suppression may reduce α-HB production [90]. In our study, the robust antioxidant abilities of ASX resulted in a substantial decrease in serum α-HB levels relative to baseline after participants were supplemented with 10 mg of ASX per day for 12 weeks. This reduction in IR is an additional contributing factor.

The results of this work complement the first conclusions reported in our doctoral dissertation “Evaluation of the effects of ASX on autophagy-related markers and study of inflammatory activity by expression of miRNAs in Type 2 diabetic patients treated with metformin [91],” as well as the matching preprint uploaded on Research Square (https://www.researchsquare.com/article/rs-4218034/v1) [92]. The dissertation set the stage for examining in individuals with Type 2 diabetes the anti-inflammatory effects of ASX by means of modulating inflammation-associated miRNAs, LPC, and α-HB. This work expands on the first results by means of thorough investigations and provides fresh understanding of the molecular basis of the possible therapeutic properties of ASX. The results provide strong evidence for prospective clinical uses since they confirm the theory that ASX supplementation can reduce diabetes-related inflammation.

This clinical trial was randomized, double-blind, and placebo-controlled, which is regarded as a robust methodological design. This design is effective in reducing bias and enhancing the validity of the results. In order to encompass a diverse array of populations with varying ages, we employed a variety of populations ranging from 20 to 60 years old in this study. Nevertheless, certain limitations may influence the broader applicability of our findings. While this study offers valuable evidence on the anti-inflammatory and metabolic benefits of ASX in T2D, certain limitations merit consideration. Although the sample size was statistically adequate, it may still constrain the generalizability of the results. Furthermore, the statistical models employed, while appropriate for the study objectives, may have intrinsic limitations in fully capturing the complexity of individual biological variability over time. The single-center setting also limits the extrapolation of findings to populations with different demographic, genetic, or environmental backgrounds. Although this study provides valuable evidence regarding the anti-inflammatory and metabolic benefits of ASX in T2D, it is recommended that future research, in order to build on these promising findings, incorporate larger multicenter studies across diverse geographical regions, adopt more comprehensive analytical approaches, and extend the duration of interventions to more thoroughly elucidate the long-term effects of ASX on diabetes-related complications.

## 5. Conclusions

In the present study, ASX exhibited the potential to attenuate inflammation in individuals with T2DM in the current study. Such effects may potentially contribute to the prevention of diabetes-related complications and reduce the risk of organ impairment. Furthermore, the administration of ASX was linked to a decrease in ACR levels, suggesting that renal function may have been enhanced. The antioxidative and antidiabetic properties of the compound may be responsible for these beneficial effects, which were also demonstrated in the reduced levels of LPC and α-HB. In particular, the findings of this investigation indicate that ASX modulates the expression of critical miRNAs associated with inflammation, such as miR-21, miR-34a, and miR-155. This regulatory effect may be a significant mechanism through which ASX exerts its anti-inflammatory effect in individuals with Type 2 diabetes. Overall, our results suggest that daily ASX supplementation may be a promising nutritional approach for enhancing IR and reducing inflammation-related complications in individuals with Type 2 diabetes. Furthermore, additional research is necessary to investigate the molecular mechanisms that underlie these effects and the broader implications of miRNA modulation in the context of metabolic disorders. Also, clinical trials involving larger and more diverse populations are recommended to comprehensively investigate the beneficial effects of ASX and to enhance the generalizability of the findings.

## Figures and Tables

**Table 1:** Effect of ASX on kidney-related markers in the ASX subgroup (patients with ACR ≥ 30).

Variables	Patients with ACR ≥ 30 mg/g (mean ± SD)		^a^ *p* value
	Baseline (*n* = 13)	After ASX (*n* = 13)	
Urea (mg/dL)	37.91 ± 9.72	29.86 ± 8.61	**0.006**
Creatinine (mg/dL)	1.34 ± 0.50	0.99 ± 0.71	**0.019**
Uric acid (mg/dL)	5.91 ± 1.48	5.77 ± 1.93	0.814
ACR (mg/g)	34.46 ± 7.36	28.39 ± 8.22	**0.003**

*Note:* Data are expressed as the mean ± SD. Bold values denote statistical significance at *p* value < 0.05. *p* values < 0.05 were considered statistically significant.

Abbreviations: ACR = albumin-to-creatinine ratio; ASX = astaxanthin; SD = standard deviation.

^a^ *p* value was reported based on the paired sample *t*-test.

**Table 2:** Differences in inflammation-related microRNA fold change expression at baseline between patients with and without albuminuria defined by ACR ≥ 30 mg/g or < 30 mg/g in the ASX subgroup.

Variables	Patients with ACR < 30 mg/g (*n* = 12)	Patients with ACR ≥ 30 mg/g (*n* = 13)	^a^ *p* value	^b^ *p* value
hsa-miR-21 (fold change)	1 ± 0.06	1.86 ± 0.38	**0.010**	0.613
hsa-miR-34a (fold change)	1 ± 0.08	2.55 ± 0.19	**0.008**	0.841
hsa-miR-155 (fold change)	1 ± 0.05	3.71 ± 0.26	**0.004**	0.916

*Note:* Data are expressed as the mean ± SD. Bold values denote statistical significance at *p* value < 0.05. *p* values < 0.05 was considered statistically significant.

Abbreviations: ACR = albumin-to-creatinine ratio; hsa-miR-21 = *Homo sapiens* microRNA-21; hsa-miR-34a = *Homo sapiens* microRNA-34a; hsa-miR-155 = *Homo sapiens* microRNA-155; SD = standard deviation.

^a^Statistical analysis was performed by independent samples *t*-test.

^b^Adjusted for baseline values, age, sex, and baseline BMI changes using the ANCOVA test.

**Table 3:** Effects of ASX on serum levels of LPC and α-HB.

Variables		The study groups		*p* value
		Placebo (*n* = 25)	ASX (*n* = 25)	
LPC (μM)	Before	138.91 ± 29.65	141.69 ± 32.59	0.812^b^
	After	144.18 ± 25.71	126.64 ± 27.93	0.004^b^
	*p* value	0.623^a^	0.005^a^	0.563^c^

α-HB (ng/mL)	Before	135.53 ± 32.26	139.50 ± 30.74	0.914^b^
	After	143.71 ± 38.59	118.72 ± 29.91	< 0.001^b^
	*p* value	0.715^a^	0.001^a^	0.744^c^

*Note:* Data are expressed as the mean ± SD. Bold values denote statistical significance at *p* value < 0.05. *p* value < 0.05 was considered statistically significant.

Abbreviations: α-HB = α-hydroxybutyrate; ASX = astaxanthin; LPC = lysophosphatidylcholine; SD = standard deviation.

^a^ *p* value was reported based on the paired sample *t*-test.

^b^ *p* value was reported based on independent samples *t*-test.

^c^Adjusted for baseline values, age, sex, and baseline BMI changes using the ANCOVA test.

## Data Availability

The data that support the findings of this study are available from the corresponding authors upon reasonable request.

## Nomenclature

α-HB: Alpha-hydroxybutyrate
ACR: Albumin-to-creatinine ratio
AMPK: Adenosine monophosphate-activated protein kinase
ASX: Astaxanthin
BMI: Body mass index
DKD: Diabetic kidney disease
FPG: Fasting plasma glucose
HbA1c: Hemoglobin A1c
HO-1: Heme oxygenase-1
hsa-miR-155: *Homo sapiens* microRNA-155
hsa-miR-21: *Homo sapiens* microRNA-21
hsa-miR-34a: *Homo sapiens* microRNA-34a
IR: Insulin resistance
LPC: Lysophosphatidylcholine
NF-κB: Nuclear factor kappa B
OGTT: Oral glucose tolerance test
PBMCs: Peripheral blood mononuclear cells
PGC-1α: Peroxisome proliferator-activated receptor gamma coactivator 1-alpha
ROS: Reactive oxygen species
T2D: Type 2 diabetes.

## Supporting Information

A part of the study's findings, which were provided for a more thorough comprehension of the main objectives of the investigation, are included in the supporting information. Additional information comprises baseline study population characteristics (refer to Supporting information Table 1) and ASX effects on biochemical parameters in the experimental groups (refer to Supporting information Table 2). Additionally, Supporting information Table 3 provides a comparative analysis of the −ΔCt values for each microRNA between individuals with ACR ≥ 30 mg/g and those with ACR < 30 mg/g. The positive correlation between inflammatory cytokines and microRNAs, including miR-21, miR-34a, and miR-155, which supports the association of these microRNAs with inflammatory processes, has also been presented in the supporting information. Additionally, the supporting information also includes the effects of ASX supplementation on proinflammatory cytokines to gain a more comprehensive understanding of its anti-inflammatory properties.
