# Correction to “Astaxanthin Modulates Inflammation in Type 2 Diabetes via Regulation of microRNAs, Lysophosphatidylcholine, and α‐Hydroxybutyrate”

**DOI:** 10.1155/ije/9845896

**Published:** 2026-01-10

A. Sharifi‐Rigi, F. Zal, M.‐H. Aarabi, M. Dehghani, N. Roustaei Rad, and S. Taghiyar, “Astaxanthin Modulates Inflammation in Type 2 Diabetes via Regulation of microRNAs, Lysophosphatidylcholine, and α‐Hydroxybutyrate,” *International Journal of Endocrinology* 2025, no. 1 (2025): 1–19, https://doi.org/10.1155/ije/5878361.

In the article, there are erroneous values in the text of Section 3.2.3. Additionally, the term “serum urea” is used to refer to Blood Urea Nitrogen (BUN) values; however, to ensure clarity and consistency, serum urea should be read as BUN.

Section 3.2.3 states the following:

“As indicated in Table 1, the baseline urinary ACR, serum urea, creatinine, and uric acid for the participants with ACR ≥ 30 mg/g in the ASX subgroup were 34.46 ± 7.36, 37.63 ± 9.72, 1.3 ± 0.05, and 5.71 ± 1.48, respectively. Consuming ASX capsules on a daily basis substantially reduced (*p* < 0.05) urinary ACR (*p* = 0.003), BUN (*p* = 0.006), Cr (*p* = 0.009), and uric acid (*p* = 0.014) to 25.39 ± 8.22, 28.01 ± 8.61, 8.4 ± 0.07, and 4.37 ± 1.93, respectively.”

Section 3.2.3 should read the following:

“As indicated in Table 1, the baseline urinary ACR, BUN, creatinine, and uric acid for the participants with ACR ≥ 30 mg/g in the ASX subgroup were 34.46 ± 7.36, 37.91 ± 9.72, 1.34 ± 0.50, and 5.91 ± 1.48, respectively. Consuming ASX capsules on a daily basis substantially reduced (*p* < 0.05) urinary ACR (*p* = 0.003), BUN (*p* = 0.006), and Cr (*p* = 0.019) to 28.39 ± 8.22, 29.86 ± 8.61, and 0.99 ± 0.71, respectively. Uric acid also decreased to 5.77 ± 1.93, although this change was not statistically significant (*p* = 0.814).”

Additionally, Table 1 should be updated to reflect the correct use of the term BUN.

**Table 1:** Effect of ASX on kidney‐related markers in the ASX subgroup (patients with ACR ≥ 30).

Variables	Patients with ACR ≥ 30 mg/g (mean ± SD)		^a^ *p* value
	Baseline	After ASX	
BUN (mg/dL)	37.91 ± 9.72	29.86 ± 8.61	**0.006**
Creatinine (mg/dL)	1.34 ± 0.50	0.99 ± 0.71	**0.019**
Uric acid (mg/dL)	5.91 ± 1.48	5.77 ± 1.93	0.814
ACR (mg/g)	34.46 ± 7.36	28.39 ± 8.22	**0.003**

*Note:* Data are expressed as the mean ± SD. *p* values < 0.05 were considered statistically significant. Bold values denote statistical significance at the *p* value < 0.05. ASX, astaxanthin.

Abbreviations: ACR = albumin‐to‐creatinine ratio, BUN = blood urea nitrogen, and SD = standard deviation.

^a^ *p* value was reported based on the paired sample *t*‐test.

The authors confirm these corrections ensure consistency throughout the manuscript and do not affect the results, discussion, or conclusions of the study.

We apologize for these errors.

