# Supplementary material for: Astaxanthin Modulates Inflammation in Type 2 Diabetes via Regulation of microRNAs, Lysophosphatidylcholine, and α-Hydroxybutyrate
Source: Int J Endocrinol. 2025 Aug 20;2025:5878361. doi: 10.1155/ije/5878361 (PMC12390588; doi:10.1155/ije/5878361)
Supplement: Supporting Information — Additional supporting information can be found online in the Supporting Information section. [file 5878361.f1.zip › Final Supplementary data.docx]

**Supplementary Description:**

A part of the study's findings, which were provided for a more thorough comprehension of the main objectives of the investigation, are included in the supplemental data. Additional information comprises baseline study population characteristics (refer to Supplementary Data Table 1) and ASX effects on biochemical parameters in the experimental groups (refer to Supplementary Data Table 2). Also, supplementary data table 3 provides a comparative analysis of the −ΔCt values for each microRNA between individuals with ACR ≥ 30 mg/g and those with ACR < 30 mg/g. The positive correlation between inflammatory cytokines and microRNAs, including miR-21, miR-34a, and miR-155, which supports the association of these microRNAs with inflammatory processes, has also been presented in the supplementary data. Additionally, the supplementary data also includes the effects of ASX supplementation on pro-inflammatory cytokines to gain a more comprehensive understanding of its anti-inflammatory properties.

**Supplementary data to:**

**Original article:**

**Astaxanthin modulates inflammation in type 2 diabetes via regulation of microRNAs, Lysophosphatidylcholine, and α-Hydroxybutyrate**

**Running title:** Effects of astaxanthin on diabetic complications.

Ali Sharifi-Rigi^1,2^, Fatemeh Zal^2^*, Mohammad-Hossein Aarabi^3^*, Nikoo Roustaei Rad^3^, Sana Taghiyar^4^

^1^Student Research Committee, Shiraz University of Medical Sciences, Shiraz, Iran.

^2^Department of Biochemistry, School of Medicine, Shiraz University of Medical Sciences, Shiraz, Iran.

^3^Department of Clinical Biochemistry, School of Pharmacy and Pharmaceutical Sciences, Isfahan University of Medical Sciences, Isfahan, Iran.

^4^Department of Clinical Biochemistry, International Campus, Shahid Sadoughi University of Medical Science, Yazd, Iran.

*** Corresponding Authors:**

1. Fatemeh Zal, PhD

Professor of Clinical Biochemistry, Department of Biochemistry, School of Medicine, Shiraz University of Medical Sciences, Postal code: 71348 45794, Shiraz-IRAN. E-mail: fatemehzal@yahoo.com

Tel/Fax: +98 713 230 30 29

2. Mohammad-Hossein Aarabi, PhD

Professor of Clinical Biochemistry, Department of Clinical Biochemistry, School of Pharmacy and Pharmaceutical Sciences, Isfahan University of Medical Sciences, Isfahan-IRAN. E-mail: [mh.aarabi@pharm.mui.ac.ir](mailto:mh.aarabi@pharm.mui.ac.ir)

**Supplementary data table 1.** Baseline characteristics of the study population.

| Variables | Placebo group | ASX group | *p*-Value |
| --- | --- | --- | --- |
| Gender (male/female) | 13/12 | 11/14 | 0.319^a^ |
| Age (year) | 50.48 ± 7.35 | 52.88 ± 7.23 | 0.462^a^ |
| Duration of diabetes (year) | 5.91 ± 2.25 | 5.97 ± 2.45 | 0.220 |
| SBP (mmHg) | 129 (118.5, 139) | 130 (120, 140) | 0.818^b^ |
| DBP (mmHg) | 79 (71, 83) | 80 (70, 82) | 0.543^b^ |
| Height (cm) | 169.97 ± 7.10 | 168.70 ± 8.12 | 0.680^a^ |
| Weight (kg) | 79.72 ± 10.21 | 81.75 ± 13.01 | 0.572^a^ |
| BMI (kg/m^2^) | 27.73 ± 9.28 | 29.54 ± 7.91 | 0.427^a^ |
| FPG (mg/dL) | 136.91 ± 19.81 | 139.84 ± 22.34 | 0.513^a^ |
| HbA1c (%) | 7.68 ± 0.76 | 7.84 ± 0.89 | 0.873^a^ |
| Urea (mg/dL) | 32.73 ± 9.25 | 34.45 ± 10.83 | 0.479^a^ |
| Creatinine (mg/dL) | 0.98 ± 0.048 | 1.1 ± 0.054 | 0.503^a^ |
| Uric acid (mg/dL) | 5.48 ± 2.03 | 5.63 ± 1.91 | 0.574^a^ |
| TG (mg/dL) | 139.87 ± 75.78 | 141.34 ± 56.00 | 0.321^a^ |
| Total cholesterol (mg/dL) | 149 ± 43.45 | 152 ± 38.15 | 0.203^a^ |
| HDL-C (mg/dL) | 48.32 ± 9.01 | 46.75 ± 7.98 | 0.809^a^ |
| LDL-C (mg/dL) | 86.31 ± 18.30 | 94.54 ± 19.97 | 0.204^a^ |
| HOMA-IR | 5.81 ± 3.74 | 4.98 ± 1.97 | 0.907^a^ |

Values are mean ± SD for data with normal distribution and median (interquartile ranges) for data not normally distributed. Statistical analysis was performed by ^a^independent samples *t*-test for parametric distributions and ^b^Mann‒Whitney U test for nonparametric distributions. *p*- Values < 0.05 were considered statistically significant. **Abbreviations:** ASX, Astaxanthin; BMI, Body mass index; BMI, body mass index; FPG, fasting plasma glucose; HbA1c, glycated hemoglobin; TG, triacylglycerol; HDL-C, high-density lipoprotein cholesterol; LDL-C, low-density lipoprotein cholesterol; HOMA-IR, homeostasis model assessment of insulin resistance; SD, Standard deviation.

**Supplementary data table 2.** Effect of ASX on biochemical parameters in the experimental groups.

| Variables |  | The study groups |  | *p*-Value |
| --- | --- | --- | --- | --- |
|  |  | Placebo (n=25) | ASX (n=25) |  |
| FPG (mg/dL) | Before  After  *p*-Value | 136.71 ± 19.64  137.18 ± 20.71  0.512^a^ | 139.27 ± 21.18  126.43 ± 18.97  **0.002^a^** | 0.081^b^  **0.008^b^**  0.850^c^ |
| HbA1c (%) | Before  After  *p*-Value | 7.61 ± 0.63  7.59 ± 0.59  0.566^a^ | 7.89 ± 0.79  7.05 ± 0.35  **<0.001^a^** | 0.414^b^  **0.004^b^**  0.318^c^ |
| HOMA-IR | Before  After  *p*-Value | 5.13 ± 1.93  5.21 ± 1.24  0.484^a^ | 5.75 ± 2.02  4.25 ± 2.37  **<0.001^a^** | 0.120^b^  **<0.001^b^**  0.622^c^ |
| Urea (mg/dL) | Before  After  *p*-Value | 33.53 ± 8.75  34.81 ± 9.46  0.212^a^ | 35.91 ± 9.63  27.50 ± 8.50  **0.009^a^** | 0.181^b^  **0.013^b^**  0.714^c^ |
| Creatinine (mg/dL) | Before  After  *p*-Value | 1.00 ± 0.45  1.13 ± 0.61  0.516^a^ | 1.17 ± 0.76  0.98 ± 0.52  0.484^a^ | 0.512^b^  0.914^b^  0.231^c^ |
| Uric acid (mg/dL) | Before  After  *p*-Value | 5.51 ± 1.48  5.76 ± 2.19  0.575^a^ | 5.68 ± 1.93  5.31 ± 1.70  0.218^a^ | 0.614^b^  0.418^b^  0.319^c^ |
| ACR (mg/g) | Before  After  *p*-Value | 27.96 ± 8.98  28.04 ± 7.50  0.514^a^ | 29.14 ± 8.30  26.93 ± 6.61  0.223^a^ | 0.213^b^  0.134^b^  0.526^c^ |
| TG (mg/dL) | Before  After  *p*-Value | 138.91 ± 48.50  139.56 ± 57.63  0. 431^a^ | 142.37 ± 52.71  131.25 ± 39.81  **0.022^a^** | 0.313^b^  **0.043^b^**  0.454^c^ |
| Total cholesterol (mg/dL) | Before  After  *p*-Value | 149.71 ± 35.86  151.88 ± 43.61  0.616^a^ | 150.98 ± 39.25  130.65 ± 36.15  **0.015^a^** | 0.713^b^  **0.011^b^**  0.619^c^ |
| LDL-C (mg/dL) | Before  After  *p*-Value | 86.47 ± 23.51  88.50 ± 35.71  0.411^a^ | 91.36 ± 28. 36  69.05 ± 36.98  **0.015^a^** | 0.213^b^  **0.022^b^**  0.950^c^ |
| HDL-C (mg/dL) | Before  After  *p*-Value | 49.58 ± 25.12  48.31 ± 22.47  0.616^a^ | 47.89 ± 21.54  51.95 ± 26.18  0.231^a^ | 0.528^b^  0.315^b^  0.498^c^ |

Data are expressed as the mean ± SD.

The ^a^*p*-Value was reported based on a paired sample *t*-test.

The ^b^*p*-Value was reported based on independent samples t-test.

^c^Adjusted for baseline values, age, sex, and baseline BMI changes using the ANCOVA test.

*p-*Values < 0.05 was considered statistically significant.

Bold values denote statistical significance at the *p*-Values < 0.05.

**Abbreviations:** ASX, Astaxanthin; FPG, fasting plasma glucose; HbA1c, glycosylated hemoglobin; HOMA-IR, homeostasis model assessment of insulin resistance; ACR, albumin-to-creatinine ratio; TG, triacylglycerol; HDL-C, high-density lipoprotein cholesterol; LDL-C, low-density lipoprotein cholesterol; SD, standard deviation.

**Supplementary data table 3.** Differences in inflammation-related microRNA expression at baseline between patients with and without albuminuria defined by ACR ≥ 30 mg/g or < 30 mg/g in the ASX subgroup.

| Variables | Patients with ACR < 30 mg/g  (n=12) | Patients with ACR ≥ 30 mg/g  (n=13) | *^a^p-*Value | *^b^p-Value* |
| --- | --- | --- | --- | --- |
| -ΔCt hsa-miR-21 | -8.23 ± 1.91 | -6.19 ± 2.03 | **0.003** | 0.613 |
| -ΔCt hsa-miR-34a | -7.96 ± 2.19 | -5.24 ± 1.43 | **0.005** | 0.841 |
| -ΔCt hsa-miR-155 | -9.98 ± 2.35 | -6.79 ± 2.64 | **0.001** | 0.916 |

Data are expressed as the mean ± SD.

^a^Statistical analysis was performed by independent samples *t*-test.

^b^Adjusted for baseline values, age, sex, and baseline BMI changes using the ANCOVA test.

*p*-Values < 0.05 was considered statistically significant.

Bold values denote statistical significance at the *p-*value < 0.05.

**Abbreviations:** hsa-miR-21, *Homo sapiens* microRNA-21; hsa-miR-34a, *Homo sapiens* microRNA-34a; hsa-miR-155, *Homo sapiens* microRNA-155; ACR, albumin-to-creatinine ratio; SD, standard deviation.

**Supplementary data table 4.** Effects of ASX on serum levels of inflammation-related markers.

| Variables |  | The study group  Placebo | ASX | ^b^p-Value |
| --- | --- | --- | --- | --- |
| TNF-α (Pg/mL) | Before  After  ^a^p-Value | 134.56 ±14.45  131.53±10.16  0.661 | 122.15±9.44  104.61±8.33  **0.008** | **0.002** |
| IL-6 (Pg/mL) | Before  After  ^a^p-Value | 109.81±21.01  108.22±7.86  0.320 | 107.83±19.95  83.46±15.84  **0.004** | **<0.001** |
| IL-1β (Pg/mL) | Before  After  ^a^p-Value | 61.82±10.21  59.53±14.08  0.178 | 62.18±15.50  46.88±16.13  **0.003** | **0.004** |

Data are expressed as mean ± SD. The ^a^*p*-Value was reported based on the paired sample *t*-test. The ^b^*p*-Value was reported based on independent samples t-test. *p-*value < 0.05 was considered statistically significant. Bold values denote statistical significance at the *p-*value < 0.05.

**Abbreviations:** ASX, Astaxanthin; TNF-α, Tumor necrosis factor-alpha; IL-6, Interleukin-6; IL-1β, Interleukin-1beta; SD, standard deviation.


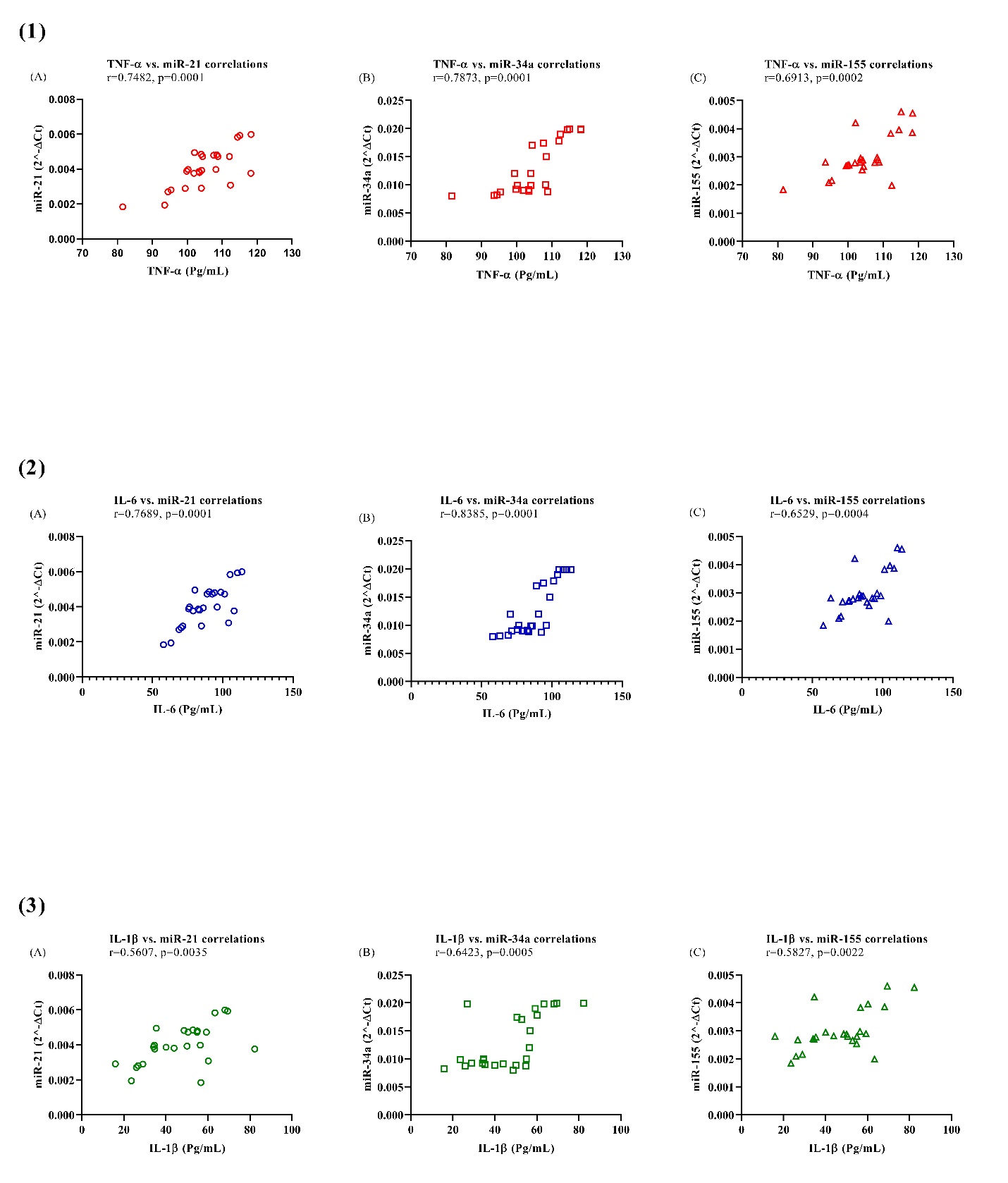


**Supplementary data figure 1.** Correlation analyses of miRNAs with Inflammatory Cytokines ( TNF-α, IL-6, and IL-1β( (1-3) in T2D patients after 12 weeks of ASX supplementation (n=25 biological replicates). Data are presented as 2^-ΔCT^ levels for miRNAs and Pg/mL for Inflammatory Cytokines.
