# Supplementary figures and images for: Astaxanthin Modulates Inflammation in Type 2 Diabetes via Regulation of microRNAs, Lysophosphatidylcholine, and α-Hydroxybutyrate
Source: Int J Endocrinol. 2025 Aug 20;2025:5878361. doi: 10.1155/ije/5878361 (PMC12390588; doi:10.1155/ije/5878361)

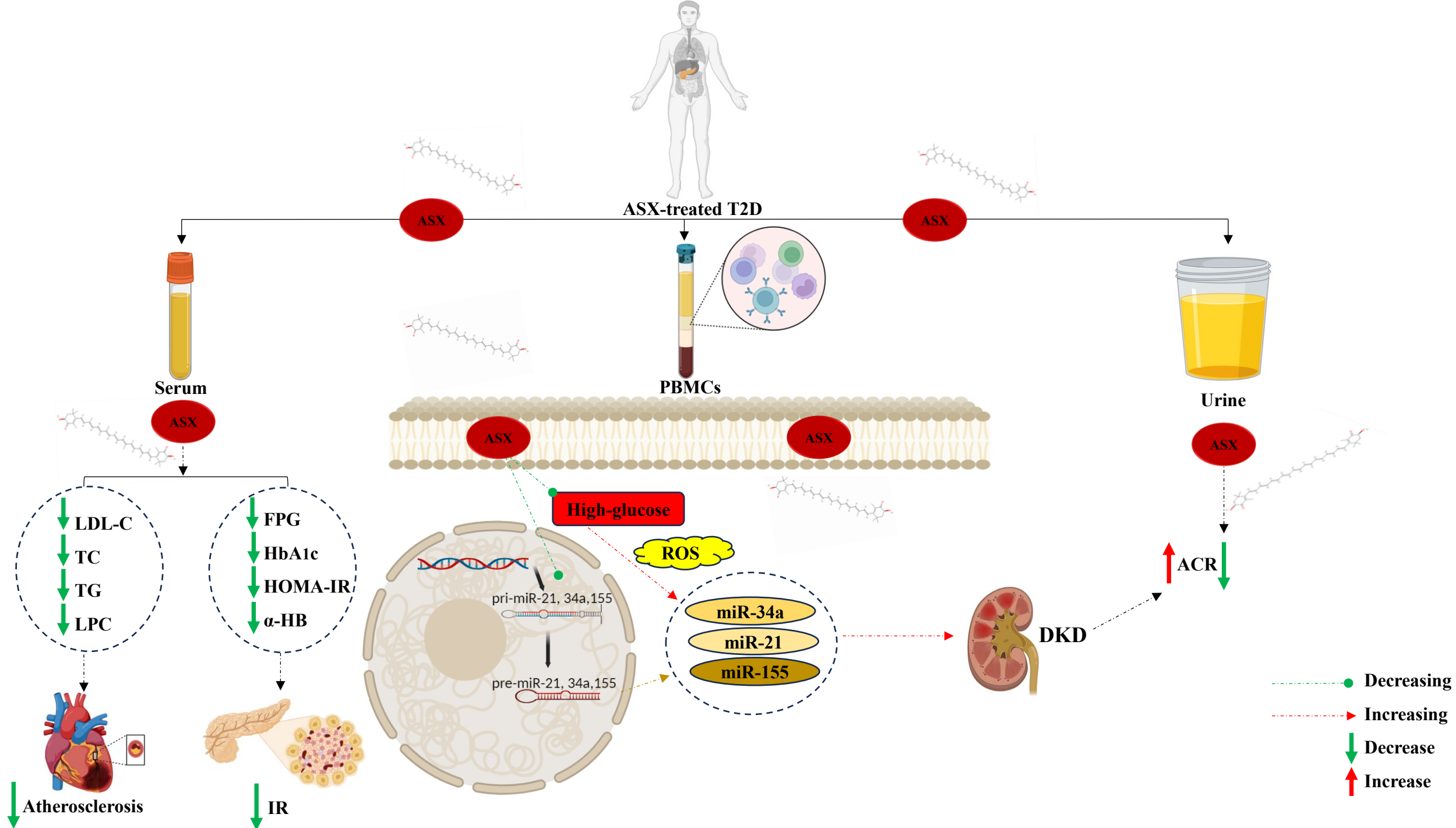

Supplement: Supporting Information — Additional supporting information can be found online in the Supporting Information section. [file 5878361.f1.zip › Graphical abstract.pdf]
